# Modulation of Autoimmune and Autoinflammatory Diseases by Gasdermins

**DOI:** 10.3389/fimmu.2022.841729

**Published:** 2022-06-01

**Authors:** Fang Liang, Weixiao Qin, Yilan Zeng, Dan Wang

**Affiliations:** ^1^ Department of Hematology, The Third Xiangya Hospital, Central South University, Changsha, China; ^2^ Department of Dermatology, The Third Xiangya Hospital, Central South University, Changsha, China

**Keywords:** gasdermins, autoimmune diseases, autoinflammatory disease, pyroptosis, inflammasome

## Abstract

Autoimmune diseases and autoinflammatory diseases are two types of the immune system disorders. Pyroptosis, a highly inflammatory cell death, plays an important role in diseases of immune system. The gasdermins belong to a pore-forming protein gene family which are mainly expressed in immune cells, gastrointestinal tract, and skin. Gasdermins are regarded as an executor of pyroptosis and have been shown to possess various cellular functions and pathological effects such as pro-inflammatory, immune activation, mediation of tumor, etc. Except for infectious diseases, the vital role of gasdermins in autoimmune diseases, autoinflammatory diseases, and immune-related neoplastic diseases has been proved recently. Therefore, gasdermins have been served as a potential therapeutic target for immune disordered diseases. The review summarizes the basic molecular structure and biological function of gasdermins, mainly discusses their role in autoimmune and autoinflammatory diseases, and highlights the recent research on gasdermin family inhibitors so as to provide potential therapeutic prospects.

## Introduction of Autoimmune and Autoinflammatory Diseases

The immune system mainly consists of innate immune system and adaptive immune system. Disorders in our immune system lead to a range of diseases, including autoimmune diseases and autoinflammatory diseases. Deficiency and/or overactivation of multiple factors within innate or adaptive immune systems may participate in the occurrence and development of these diseases, such as experimental autoimmune encephalomyelitis (EAE), Inflammatory bowel disease (IBD), psoriasis, etc. (1). During the occurrence of autoimmune diseases, the immune system is out of balance and immunocytes are overactive, the release of cytokines further lead to abnormal secretion of autoantibodies, resulting in inflammation and damage to tissues and organs (2, 3). While the progression of the diseases can be accelerated because of other key factors, such as gene, environment, hormones, and so on. In 1999, the genetic mechanism of a rare familial disease characterized by recurrent inflammatory episodes which are defined as tumor necrosis factor receptors associated periodic fever syndrome (TRAPS) was discovered. Another Familial Mediterranean Fever (FMF), an autosomal recessive hereditary disease characterized by recurrent peritonitis. TRAPS and FMF are both autoinflammatory diseases (4, 5). Gill et al. envisaged autoinflammatory diseases as counterparts to autoimmune disorders, and proposed that autoinflammatory diseases are related to innate immune system dysfunction and are determined by genes (1). McGonagle and McDermott proposed that dysfunction of the innate immunity or the adaptive immune system is the differentiated element of autoimmune diseases and autoinflammatory diseases (6). Approximately, disorders of innate immune system represent autoinflammatory diseases, while disorders of adaptive immune system reflect autoimmune diseases. However, innate and adaptive immunities are inextricably intertwined and influence each other (7).

Autoimmune diseases afflict nearly 5%-8% of the population worldwide and represent a major global socioeconomic issue (8, 9). So far, there are no fewer than 80 autoimmune diseases have been discovered which are divided into systemic or organ-specific. Systemic Lupus Erythematosus (SLE) and Central Nervous System (CNS) reflect the systemic autoimmune diseases, while type 1 diabetes images the organ-specific autoimmune diseases such as the pancreas (9, 10). During such diseased conditions, the immune system is out of balance, immunocytes are overactive, and there is imbalance of cytokine which further leads to abnormal secretion of autoantibodies, resulting in inflammation and damage to tissues and organs (11). At this stage anti-inflammatory and immunosuppressive therapy are commonly used in the treatment of autoimmune diseases to the symptoms (12–16). Because of the complex mechanisms involved in the pathogenesis of the autoimmune diseases, there is yet no effective treatment available, and therefore, it is important to understand these processes.

Autoinflammatory diseases are characterized by antigen-independent-immune pathways overactivation arising primarily inflammation. Common autoinflammatory diseases include FMF, TRAPS, Cryopyrin-associated periodic syndrome **(**CAPS) and Mevalonate kinase deficiency (MKD), but not all autoinflammatory diseases manifest in their canonical forms. Hyperactivity of proinflammatory cytokine is found to mediate and participate in autoinflammatory diseases. FMF was identified as the first autoinflammatory disease, which is an inflammasomopathy that arises from the MEFV gene mutations, coding pyrin (expressed predominately by innate lineages) (4, 17). Other autoinflammatory diseases mostly arise out of NF-κB and/or aberrant TNF activity, interferon production and complement activation, and excessive interleukin-1 (IL-1) signaling (7, 18, 19). So far, IL-1 blockers have been approved for a variety of autoinflammatory diseases. New categories of autoinflammatory disease and more treatment will emerge over time.

Autoimmune diseases and autoinflammatory diseases are difficult to cure and seriously affect the quality of life of patients which involve various pathogenic mechanisms such as genes, immunity, inflammation, etc. Therefore, the existing research is summarized to deepen the understanding of the disease and provide help for the diagnosis and treatment of those diseases.

## Gasdermin Family, Pyroptosis and Inflammasomes

As early as in 2000s, the gasdermin family was first discovered as a gene family associated with corneal opacity in mice (20, 21). The gasdermin family is predominantly expressed in immune cells, gastrointestinal tract, and skin and linked with many diseases including autoimmune diseases and autoinflammatory diseases (**Table 1**). This family consists of six genes in humans, GSDMA through GSDMF. In mice, it encodes ten members, which respectively are GSDMA1 to GSDMA3, GSDMC, GSDMC2 to GSDMC4, GSDMD, GSDME, and DFNB59 (22, 23). All of these members possess a semblable molecular structure except DFNB59. A pore-forming N-terminal domain and a regulatory C-terminal domain connected by highly variable and flexible linking regions compose the nuclear structure, which is thought to be necessary for GSDM activation (24). The N-terminal domain (GSDM-NT) is required for forming pores in membranes while the auto-inhibitory C-terminal domain (GSDM-CT) is asked to keep its inactivation (24, 25). Structurally, GSDM-NT predominantly contains loops and β-strands, and sustains drastic conformational changes in pore formation (24). Nevertheless, GSDM-CT is nearly α-helical and remains a compact globular conformation. GSDM-CT forms electrostatic, hydrophobic, and hydrogen bonding interactions after folding back on GSDM-NT (24–26). *In vitro*, GSDM-NT and GSDM-CT keep bound without lipids, even if the interdomain container is cleaved, this finding suggests that the lipid may play a critical role in the separation of GSDM-NT from GSDM-CT. In simple terms, lipid binding, oligomerization, and membrane insertion make up the pore formation of GSDM (25, 27).

**Table 1:** Gasdermin family members, functions, and disease correlation.

Human GSDM	Gene location	Predominant expression	Biological function	Associated disease
GSDMA	17q21	esophagus, stomach, skin, mammary gland	Apoptosis cell proliferation	IBD, SSc, RA, asthma in children
GSMDB	17q21	immune cells, airway, liver, gastrointestinal epithelial, neuroendocrine	pyroptosis	IBD, asthma, type I diabetes.
GSDMC	8q24.1-8q24.2	colon, spleen, trachea, esophagus, caecum, small intestine	Not known	Not known
GSDMD	8q24.3	skin, immune cells, astrointestinal tissue	Pyrotpsis, caspase-1, LPS-activatedcaspase-11,	EAE, FMF, NOMID
GSDME	7p15	brain, heart, kidney, cochlea, placenta	Apoptosis, pyroptosis	Hearing loss, several inflammatory skin diseases

**Figure 1:**
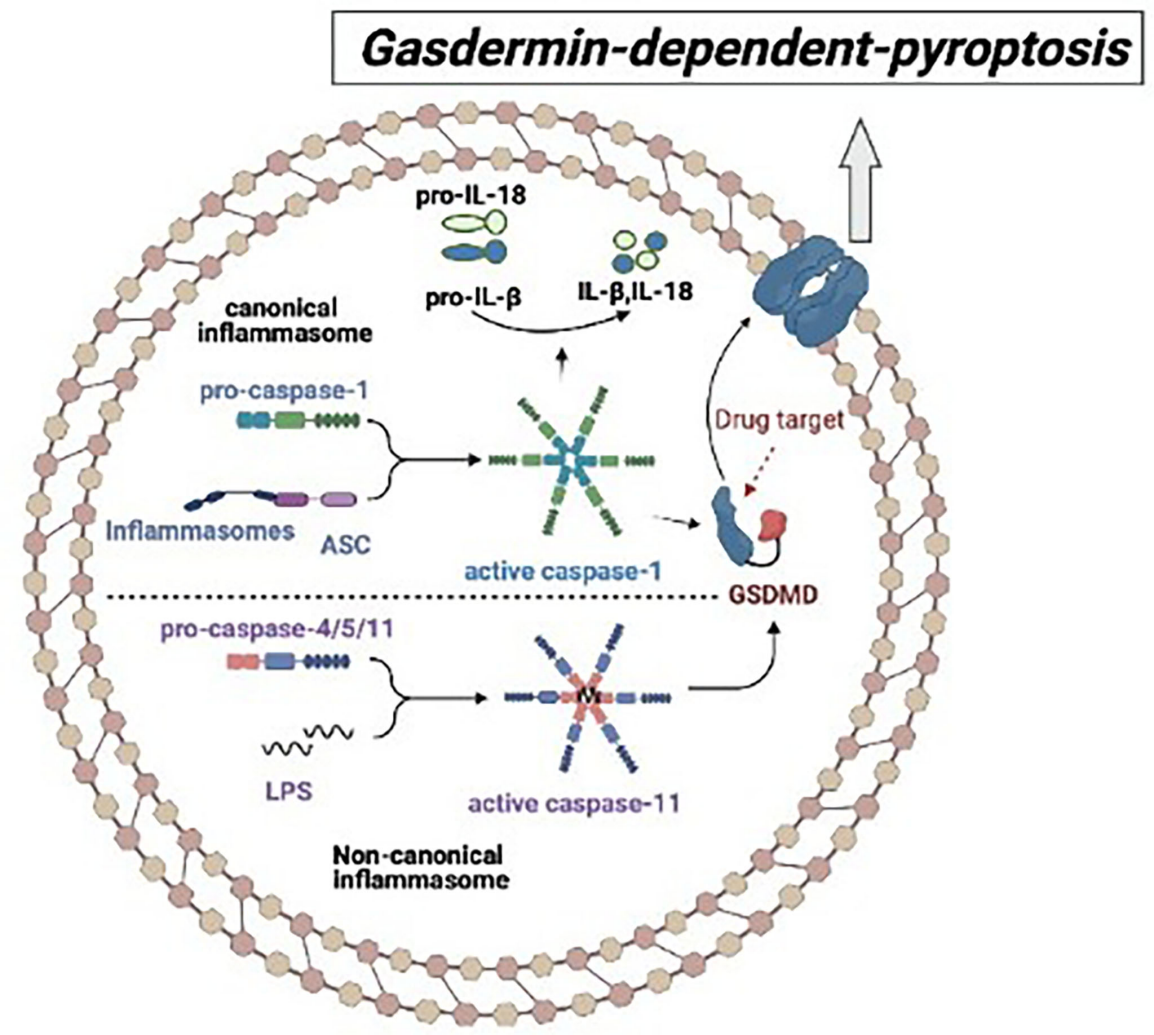
The mechanism of gasdermin-dependent-pyroptosis: In the canonical pyroptosis pathway, ASC recruits intracellular pro-Caspase-1 to bind to the inflammatory complex, and then pro-caspase-1 auto-cleaves into activated Caspase-1, which cleaves GSDMD and promotes IL-1β and IL-18 to maturate. In the non-canonical pyroptosis pathway, inflammatory stimulators such as lipopolysaccharide (LPS) directly bind to Caspase-4/5/11 and cause oligomeric activation of Caspase-4/5/11, activated Caspase-4/5/11 cleaves GSDMD and triggers pyroptosis.

Pyroptosis is a kind of programmed cell death (PCD) associated with inflammation, which plays an important role in autoimmune and autoinflammatory diseases (28, 29).. Pyroptosis is a protective host defense that protect the cell from intracellular infection by removing the injured cells and concurrently triggering an inflammatory response that executes the following activation of caspase-1 or caspase-11 (21). Activated caspase-1 or caspase-11 cleaves GSDMD independently, releasing the N-terminal fragment (GSDMD-NT) associated with the cytoplasm membrane, which forms the cell membrane pores by oligomerization. Then, the cell swelling causes the membrane lysis and finally leads to pyroptosis (**Figure 1**) (30–33).

Inflammasome plays a crucial role in the process of pyroptosis. Inflammasomes are cytosolic protein complexes consisting of sensor, adaptor, and effector which are whole protein that is stimulated by pathogen-associated molecular patterns (PAMPs) and endogenous danger-associated molecular patterns (DAMPs) (34). Upon activation by diverse danger signals, members of the NOD-like receptor (NLR) and pyrin and HIN domain-containing (PYHIN) protein families as sensors to combine with apoptosis-associated speck-like protein containing a caspase recruitment domain (ASC) adaptor, subsequently recruit effector caspase-1 to form canonical inflammasome so as to initiate a downstream signal. Subsequently, the activated caspase-1 cleaves pro-inflammatory cytokines interleukin 1β (IL-1β) and interleukin 18 (IL-18), allowing them to mature and secret (35, 36). The non-canonical inflammasome is activated by bacterial cell wall component lipopolysaccharide (LPS), and executed by caspase-11 (human caspase-4/5) (30, 31, 37–39). Therefore, both canonical and non-canonical inflammasome participate in the occurrence of pyroptosis by cleaving GSDMD into GSDMD-NT so as to form a pore in the cell membrane (**Figure 1**).

Gasdermin family is well known to play a vital role in pathogen infection, while more and more studies show that both pyroptosis and its related inflammatory cytokines IL-1β and IL-18 are the key factor in the process of non-infection related diseases. IL-1β induces vasodilation, inflammation and immunity extravasation, and also takes part in formation of adaptive immune responses. IL-18 promotes natural killer (NK) cells, T helper 1 (TH1) cells and cytotoxic T cells to produce interferon-γ (IFN-γ) in these cells, and promotes T helper 2 (TH2) cells maturation, and triggers local inflammation. These findings suggest that gasdermins, and autoimmune as well as autoinflammatory disease are inextricably linked (40–43). Therefore, the following section will focus on introducing the gasdermin family and summarizing the role of gasdermins in autoimmune and autoinflammatory diseases.

## The Roles of Gasdermins in Autoimmune and Autoinflammatory Diseases

Multiple functions of pyroptosis and inflammasomes are identified in various pathophysiological conditions, and their role in autoimmune and autoinflammatory diseases have been extensively studied recently. Therefore, we will summarize the relation between each subtype of the gasdermin family (GSDMA, GSDMB, GSDMC, GSDMD, GSDME) and autoimmune and autoinflammatory diseases.

## GSDMA

The human GSDMA gene is located at 17q21. It is widely expressed in the epithelial cells of the esophagus, stomach, skin and mammary gland, but is often not expressed in primary and the gastric cancer cell lines. It has been suggested that GSDMA suppresses the growth of human gastric epithelial pit cells as it possibly takes part in a regulatory pathway for apoptosis (44). The high expression of GSDMA in non-IBD colonic, noninflamed samples was linked with the IBD susceptibility allele (rs2872507) (45). In childhood asthma, there was a significant correlation of GSDMA (rs7212938) with it as GSDMA may drives the frequency of asthma (46). In addition, GSDMA has been reported to carry rs3894194, which is rich in gene, and regarded as being linked with certain immune diseases such as IBD, asthma and rheumatoid arthritis (RA) (47). Aida et al. reported that the function of r3894194 in systemicsclerosis (SSc) could be mediated by GSDMA expression in macrophages. They found that GSDMA was upregulated in SSc monocyte-derived macrophages, and was an important expression quantitative trait locus (e-QTL) in interferon gamma (IFN-γ) or LPS-triggered monocytes and SSc macrophages (48). Overall, GSMDA is linked with several autoimmune diseases, while the exact pathogenesis still remains blurred, GSDMA may affect those diseases *via* effects on apoptosis and cell proliferation. It warrants further experimental investigation.

## GSDMB

GSMDB is located at 17q12 in the human chromosome (22, 49). It clusters with GSDMA (50). GSDMB is also broadly expressed in the immune cells, airway, gastrointestinal epithelial, liver, and neuroendocrine (51–53). Genome-wide association studies (GWAS) implicated that the polymorphisms in GSDMB were markedly associated with IBD, asthma, and type I diabetes. The minor allele A of rs2872507 in GSDMB was the risk allele for RA and IBD (Crohn’s disease and ulcerative colitis) but was a protective allele for asthma (47). While another study found that the levels of GSDMB were increased remarkably in patients with asthma, and were associated with the severity of asthma (54). A recent study showed that GSDMB was increased in intestinal epithelial cells (IECs) in IBD, and it participated in the regulation of cell proliferation, migration, and adhesion rather than mediating pyroptosis. GSDMB-linked IBD single-nucleotide polymorphisms (SNPs) hinder epithelial recovery/repair (55). Moreover, Luke Jostins et al. reported that numerous IBD loci also contributed to immune diseases (most obviously with ankylosing spondylitis and psoriasis) (56). Those consequences indicate that GSDMB increases the risk for various autoimmune diseases, but the detailed molecular mechanisms remain unclear. There was reported that the GSDMB can bind with caspase-4 to promote non-canonical pyroptosis and facilitate the GSDMD cleavage. This may be associated with the pathogenesis of autoimmune diseases (57).

## GSDMC

GSDMC gene has been located at 8q24.1-8q24.2 and is highly expressed in colon, spleen, trachea, esophagus, caecum, and small intestine (38, 49, 58). There are few studies on GSDMC. Lumbar disc degeneration (LDD) mainly manifests as low back pain, disc herniation, spinal stenosis, spinal instability, and radiculopathy. A recent study suggested that autoimmunity may be possible pathogenesis of this disease (59). GWAS in a Chinese population reported that GSDMC was associated with lumbar disc degeneration (LDD). In patients with LDD, the mRNA level of GSDMC was apparently increased and the plasma expression level of GSDMC was also apparently upregulated which were linked with the rs6651255 and rs7833174 (60). GSDMC was also expressed in skin keratinocytes, and the UV radiation increased its expression by triggering NFATc1 signaling (61, 62). Nevertheless, the mechanism of GSDMC in other autoimmune and autoinflammatory diseases is not yet well-understood.

## GSDMD

The GSDMD gene is located at 8q24.3 in the human chromosome. It has been extensively studied for decades. As the key substrate of caspase-dependent-pyroptosis, GSDMD was widely expressed in skin, immune cells (especially macrophages and dendritic cells), and gastrointestinal tissue (63). Approximately 480 amino acids constitute GSDMD, which is in two domains: the N-terminal (GSDMD-NT) and the C-terminal (GSDMD-CT). GSDMD is cleaved by activated caspase-1, LPS-activated caspase-11 (human caspase-4/5) to form pore-forming GSDMD-NT (31). The pores of GSDMD-NT can bind with phosphatidylserine and phosphatidylinositol phosphates, causing cell lysis and releasing of IL-1β, IL-18, and HMGB1. GSDMD-NT also combines with cardiolipin in the bacteria membranes, and kills them quickly. In addition, neutrophil elastase (ELANE) and cathepsin G are both able to activate the GSDMD (24, 64–66).

Recent studies have shown that GSDMD is involved in the process of immune and inflammatory responses. Catherine R et al. reported that AIM2 inflammasome took part in neurodevelopment through regulating GSDMD activation instead of IL-1 and/or IL-18 production. In this study, the DNA damage-induced cell death was significantly reduced in CNS cells lacking GSDMD or caspase-1/11, which means that GSDMD activation may hinder neurodevelopment (67). Sheng Li et al. firstly demonstrated that GSDMD was necessary for EAE and could promote neuroinflammation and demyelination (68). During impaired neurodevelopment, a large amount of DNA damage is produced, which may immunologically activate signals that can cause autoimmune diseases of the nervous system. Another research provided that TPPU (a potent soluble epoxide hydrolase) can attenuate chronic EAE by suppressing caspase-11 and GSDMD in the CNS of EAE mice, a model of multiple sclerosis (MS) (69). Dataset analysis showed a significant elevation of caspase-4/5, pro- IL-1β, and GSDMD in human psoriatic lesions. Besides, caspase-11 contributed to the pathogenesis of imiquimod-induced psoriasis in mice through the mediation of GSDMD activation and cell death (70). The process of GSDMD-dependent pyroptosis also took part in RA through the releasement of inflammatory mediators (71). Another research found that inhibition of the caspase-11 (executor of pyroptosis) could enhance mesenchymal stromal cells (MSCs) efficacy in inflammatory diseases, such as IBD and EAE. MSCs present immunosuppressive properties and were currently being used as promising treatments for various inflammatory diseases, including MS (46, 72, 73). A report demonstrated that the pathogenesis of FMF, an IL-1β-dependent autoinflammatory disease, relies on the GSDMD-dependent-pyroptosis (74). Neonatal-onset multisystem inflammatory disease (NOMID) is the most severe phenotype in cryopyrinopathies (a spectrum of autoinflammatory disorders), and there has been reported that all NOMID-related inflammatory symptoms were prevented in GSDMD-deficient NOMID mice compared to wildtype NOMID mice (75). Overall, these findings suggest that activation of GSDMD aggravates the pathogenesis of various diseases induced by inflammasomes. Targeted GSDMD may provide new perspectives for the treatment of autoimmune and autoinflammatory diseases.

## GSDME

GSDME is located at 7p15 in the human chromosome. It was defined as a mutated gene (DFNA5) firstly that was associated with progressive hearing loss (76–83). Subsequent studies found that the DFNA5 might be crucial to chondrocyte development. GSDME is widely expressed in brain, heart, kidney, cochlea, and placenta (76, 84). Similar to GSDMD, GSDME plays a critical role in apoptosis and pyroptosis (85, 86). GSDME releases N-terminal fragment (GSDME-NT) after its cleavage and activation by caspase-3 (27). In addition to forming pores and triggering necrosis or pyroptosis, GSDME-NT permeabilizes the mitochondrial membrane, leading to release cytochrome C, and eventually enhances the activation of caspase-3 during apoptosis (25).

Several studies revealed a link between GSDME and immunity/inflammation. Oncostatin M (OSM), an IL-6 family member, has been found to be overexpressed in several inflammatory skin diseases [including keloid (87), scleroderma (88, 89), and psoriasis (90, 91)]. The level of GSDME in human keratinocytes (HaCaT cells) was up-regulated, while OSMR knockout blocked the upregulation of GSDME. Besides, GSDME knockout inhibited OSM-induced keratinocyte pyroptosis. This study suggested that GSDME played a role in OSM-mediated inflammation (92). Another study found that GSDME-mediate pyroptosis was critical for the trigger of renal tubule injury induced by ureteral obstruction and promoted kidney inflammation and fibrosis (93). Overall, GSDME has pro-inflammatory functions and may be involved in the pathogenesis of more inflammatory diseases.

## Therapy Targeting of Gasdermin Family

Based on the important role of gasdermin family in autoimmune and autoinflammatory diseases, this study further summarizes the inhibitors targeting gasdermin family to provide new prospection for disease treatment.

Among all subtypes of the gasdermin family, inhibitors targeting GSDMD have been revealed the most due to its rich biological functions. Necrosulfonamide (NSA) was originally proved to be an inhibitor of necrosis by targeting the Cys86 Residue of MLKL to reduce MLKL-dependent pore formation and death (94). In the work of *Joseph K. Rathkey’s* group, they found that NSA inhibited pyroptosis and IL-1β release by binding to GSDMD and blocking p30-GSDMD oligomerization *in vitro*, and NSA did not block GSDME-dependent pyroptosis and the upstream inflammasome activation (95). NSA promoted survival rate and decreased inflammatory cytokine level of sepsis model *in vivo (* 95). Conversely, another study showed that NSA blocked canonical NLRP3 inflammasome priming and activation so as to reduce pyroptosis (96). Both studies showed that NSA inhibited GSDMD-dependent pyroptosis to play a role in treating diseases by two different mechanisms, suggesting that NSA may provide a theoretical basis for the treatment of GSDMD-related inflammatory diseases. Besides, Jun Jacob Hu et al. discovered that disulfiram, a drug used to treat alcohol addiction, inhibited GSDMD-mediated pyroptosis rather than other subtypes of gasdermin family. Disulfiram targeted Cys191/Cys192 in GSDMD so as to inhibit pore formation and IL-1β release, and protected against septic death in mice (97). In another study, LDC7559 from a chemical library was found to inhibit GSDMD-dependent pyroptosis and IL-1β release so as to reduce neutrophil extracellular traps (NETs) formation (98). Activated macrophages present as the Warburg effect, which is characterized as a metabolic change from glycolysis to aerobic glycolysis and the accumulation of Krebs’ cycle intermediates. The alteration of metabolism further impacts the immune state of macrophages (99). Fumarate (DMF), an intermediate of Krebs’ cycle, blocked GSDMD oligomerization and the related pyroptosis by inducing S-(2-succinyl)-cysteine of GSDMD. Dimethyl fumarate with the cell-permeable ability, reduced septic shock and familial Mediterranean fever, and experimental autoimmune encephalitis (100).

Collectively, several studies have found various inhibitors of gasdermin family (mainly GSDMD), and they can improve a variety of diseases. Although these diseases are mainly sepsis, there is also involved to autoimmune and autoinflammation diseases.

## Conclusions

Our knowledge of gasdermins function becomes increasingly extensive based on a large of recent studies. Most subtypes of gasdermin play a pro-inflammatory role in the autoimmune and autoinflammation diseases, including IBD, RA, SSc, EAE, psoriasis, etc. Among the whole types of gasdermin, GSDMD is the most studied subtype of gasdermins due to its tight correlation with inflammasomes activation and the related inflammatory cytokines release. Mice GSDMD deficiency presents a protection role in certain autoinflammatory diseases, which suggests that the gasderim family prospects be the key target for the treatment of autoimmune and autoinflammation diseases. Targeted GSDMD has also developed a variety of inhibitors, which are expected to be used for clinical treatment. Besides, GSDME is crucial in several complicated inflammatory skin diseases which are both autoimmune and autoinflammation related, such as psoriasis, keloid, etc.

Although there are more and more researches on gasdermin family in autoimmune and autoinflammation diseases, there are still a lot of unanswered research questions out there: whether GSDMD deficiency or inhibitor play a protective role in various immune-related diseases? Whether targeting GSDME improve the progression of immune disordered diseases? GSDMA and GSDMB have been shown to be important in IBD, whether other family members also play important roles in IBD? At present, the research of GSDMC is relatively lacking, and further discussion is required. To sum up, better understanding of the gasdermin family and exploring targeted inbitors may improve treatment efficiency of autoimmune diseases, and autoinflammatory diseases.

## Author Contributions

WQ and FL, writing, reviewing and editing. FL, contributed a lot during the reviewing.

## Funding

This study was supported by Longitudinal subject of Municipal Science and Technology Bureau (grant number XY050033).

## Conflict of Interest

The authors declare that the research was conducted in the absence of any commercial or financial relationships that could be construed as a potential conflict of interest.

## Publisher’s Note

All claims expressed in this article are solely those of the authors and do not necessarily represent those of their affiliated organizations, or those of the publisher, the editors and the reviewers. Any product that may be evaluated in this article, or claim that may be made by its manufacturer, is not guaranteed or endorsed by the publisher.
